# Therapeutic positioning of asciminib in chronic myeloid leukemia patients previously treated with multiple tyrosine kinase inhibitors in Qatar

**DOI:** 10.1007/s12672-025-03759-7

**Published:** 2025-10-23

**Authors:** Rola Ghasoub, Anas Hamad, Susanna El Akiki, Shehab Fareed, Anil Ellahie, Omar Ismael, Mohamed A. Yassin

**Affiliations:** 1 Department of Pharmacy, National Center for Cancer Care and Research, Doha, Qatar; 2 Diagnostic Genomic Division, Hamad Medical Corporation, Doha, Qatar; 3 Department of BMT & Hematology, NCCCR, Doha, Qatar

**Keywords:** Leukemia, myeloid, chronic phase, Asciminib, Tyrosine kinase inhibitors, Cardiovascular diseases, Safety, Middle east

## Abstract

Chronic phase-chronic myeloid leukemia (CP-CML) in Qatar presents unique epidemiological and clinical challenges compared with those in the Western population, including nearly half of the affected patients being under 40 years of age. Additionally, the high prevalence of cardiovascular comorbidities in this region significantly influences treatment decisions. This position statement presents an evaluation of the therapeutic role of asciminib in patients previously treated with two or more tyrosine kinase inhibitors and its relevance within Qatar’s healthcare system. Studies have demonstrated asciminib’s superior efficacy in achieving higher rates of major molecular response than bosutinib. Furthermore, compared with ponatinib, asciminib offers a favorable cardiovascular safety profile, making it a preferred option for patients at risk of cardiovascular complications. Cost-effectiveness analyses further support its adoption as a viable treatment alternative in Qatar. Given these considerations, asciminib emerges as a promising therapeutic option for patients with CP-CML requiring third-line treatment, striking a balance between efficacy, safety, and economic feasibility within the local healthcare framework.

**Supplementary Information:**

The online version contains supplementary material available at 10.1007/s12672-025-03759-7.

## Introduction

Chronic myeloid leukemia (CML) is a myeloproliferative neoplasm characterized by myeloid hyperplasia. The proliferating cells present with an acquired cytogenetic abnormality (Philadelphia chromosome (Ph) and/or *BCR::ABL1* rearrangement) that results in a fusion gene encoding an oncoprotein with a constitutively active tyrosine kinase domain [1]. The natural course of the disease is triphasic, consisting of an initial indolent chronic phase (CP), an accelerated phase (AP), and a blast phase (BP). Most patients are diagnosed in the CP. If CP-CML is not adequately managed with available therapies, it may progress to AP or BP, which are associated with poorer prognosis and limited treatment options [1].

Worldwide, CML is a rare disease, with an incidence of 1–1.5 cases per 100,000 inhabitants per year and is most commonly diagnosed between the ages of 50 and 60 years. Although prevalence at younger ages is rare, CML can be more aggressive in adolescents and young people [2–4]. The prevalence of CML has increased in recent years primarily due to the use of tyrosine kinase inhibitors (TKIs) and advancements in diagnostic capabilities, which have extended survival rates from 5 to 7 years to nearly normal life expectancy [1]. In Qatar, the age-standardized incidence rate of CML is 0.38 per 100,000 people [5]. Notably, the CML population in Qatar is younger than that in Western countries, with a single-center study at the National Center for Cancer Care and Research (NCCCR) revealing that 49.11% of CML patients in Qatar were under 40 years of age [6, 7]. A survey of CML management practices in the Gulf region, including Qatar, further confirmed this observation of a younger age of CML diagnosis than that in Western populations [8].

The general approach to CML management should account for multiple factors, including comorbidities [9]. Diabetes, hypertension, and hyperlipidemia are highly prevalent in the population of Qatar. In a population-based study of 1117 participants from primary healthcare centers in Qatar, 33.6% had hypertension, 22.9% had elevated triglyceride levels, 35.9% had high cholesterol levels, 25.3% had low high-density lipoprotein levels, and 20.4% high low-density lipoprotein levels [10]. Due to the high prevalence of these comorbidities, the Qatari population is at an increased risk of cardiovascular disease (CVD), which is the leading cause of non-communicable disease-related deaths in the country, accounting for 27% of all deaths [11]. Furthermore, even CML patients reflect this high prevalence of diabetes, hypertension, and hyperlipidemia at baseline, which may adversely impact CML survival rates [12]. Imatinib, dasatinib, bosutinib, and nilotinib are the four TKIs approved for first-line treatment. Ponatinib is recommended for patients with resistance or intolerance to prior TKIs. Asciminib, initially used as a third-line therapy, is now also approved for second-line (patients previously treated with one TKI) and as upfront therapy for patients with CP-CML [13, 14]. However, considering the presence and risk of comorbidities, a personalized approach that takes comorbidities into account, including an assessment of cardiovascular risk before initiating TKI therapy, is essential in Qatar [12, 15].

Qatar, with a population of approximately 3 million and a median age of 32 years, has rapidly developed its healthcare system over the past 30 years in both the public and private sectors. The Hamad Medical Corporation (HMC), the primary governmental healthcare provider, serves approximately 80% of patients in Qatar. The NCCCR in Doha, part of the HMC, is the premier cancer hospital in Qatar [16]. This position paper aims to evaluate the role of asciminib in the treatment of CP-CML in patients previously treated with two or more TKIs in Qatar, considering regional evidence, international guidelines, and the specific characteristics of Qatar’s patient population and healthcare system [8, 17, 18].

## Methodology

CML experts from Qatar were involved in developing a position statement, ensuring that global evidence is contextualized to Qatar’s healthcare system and clinical practice. To develop this position statement, a comprehensive and evidence-based approach was undertaken through a systematic literature review. PubMed, EMBASE, and the Cochrane Library were searched for articles published between January 2010 and March 2024 using key terms such as “chronic myeloid leukemia” or “CML,” “asciminib” or “STAMP inhibitor,” “tyrosine kinase inhibitor” or “TKI,” “third-line therapy” or “resistant CML,” “Middle East,” and “cardiovascular risk” or “comorbidities,” along with “cost-effectiveness” or “healthcare resource utilization.” The search strategy is presented in Supplementary Table S1. Eligible studies included English language publications of randomized controlled trials, observational studies, systematic reviews, meta-analyses, and guidelines focusing on adult CML patients and addressing the efficacy, safety, or cost-effectiveness of asciminib or other third-line CML therapies.

The following sections discuss key aspects of CML management, including the evolving TKI landscape, the role of genomic testing for accurate diagnosis and treatment decisions, HMC’s testing strategy, an overview of asciminib and its pharmacology, its efficacy and safety profile, guideline recommendations, real-world evidence, and cost-effectiveness considerations.

## CML and its management

### TKI treatment landscape in CML management

Historically, the primary objective of CML treatment was to improve overall survival (OS), which has been largely achieved with the available TKI therapies. Current treatment goals have expanded to include achieving deep molecular response (DMR), treatment-free remission (TFR), improved tolerability to treatment, and enhanced patient quality of life (QoL) [19]. For newly diagnosed CP-CML, treatment options include first-generation TKI imatinib and second-generation TKIs nilotinib, dasatinib, and bosutinib. Of these, imatinib remains the most widely used TKI [20, 21]. In the Gulf region, including Qatar, a recent survey showed that imatinib is the most common first-line treatment, followed by second-generation TKIs [8].

The effectiveness of treatment is measured by both cytogenetic response (reduction in Ph-positive metaphases in the bone marrow) and molecular response (decrease in *BCR::ABL1* transcripts in the peripheral blood) [22, 23]. The European LeukemiaNet (ELN) and National Comprehensive Cancer Network (NCCN) guidelines outline optimal response milestones for CML treatment, defining early molecular response as *BCR::ABL1* ≤ 10% at 3 months and a response of ≤ 1% at 6 months and major molecular response (MMR) as *BCR*::*ABL1* ≤ 0.1% at 12 months [20, 24]. Achieving these milestones is associated with better long-term outcomes and an increased likelihood of successful TFR attempts. To ensure effective treatment response and guide clinical decisions, regular molecular monitoring using standardized real-time quantitative polymerase chain reaction (RT-qPCR) is essential [25].

Despite advances in TKI therapy, some patients continue to experience primary or secondary CML treatment resistance or develop intolerance to these therapies. Mutations in the *BCR::ABL1* kinase domain, particularly T315I, are associated with increased resistance and disease progression [26–28]. Following first-line treatment intolerance or failure, observed in approximately 50% of patients, second-generation TKIs are typically preferred. The choice of TKIs depends on the first-line treatment, disease phase, reason for therapeutic failure, presence of kinase domain mutations, patient comorbidities, and the safety profile of each TKI [1, 9, 21, 29]. A Gulf region survey indicated that dasatinib (44%) was the most common second-line treatment, followed closely by nilotinib (36%) [8]. The failure rates in second- or third-line treatments are 60%–70%. For patients with suboptimal response after two or more TKIs, allogeneic hematopoietic stem cell transplantation is considered, though it carries high morbidity and mortality risks [20, 23]. As treatment goals evolve, achieving and maintaining DMR has become crucial for attempting TFR. While TFR is an emerging goal, particularly for younger patients, data on long-term outcomes and optimal strategies are still being gathered [20]. TFR was considered the most important long-term treatment objective by all respondents in the Gulf region expert survey [8].

Mutations in the kinase domain of *BCR::ABL1* are associated with TKI resistance [27]. In Qatar, mutations conferring resistance to imatinib have been reported, with one study attributing 50% of treatment failures to additional chromosomal abnormalities [30]. Mutational analysis of the *BCR::ABL1* kinase domain typically involves genomic sequencing and is recommended in patients with a loss of response to treatment or at diagnosis for patients in the AP or BP [27, 28].

### Importance of genomic testing for accurate diagnosis and informed management

Treatment of CML in the Gulf region, including Qatar, aligns with global standards and relies on genomic testing for accurate diagnosis and *BCR::ABL1* messenger ribonucleic acid (mRNA) transcript levels monitoring [8]. Treatment objectives include achievement of an early molecular response, prolonged OS, and the possibility of TFR as an alternative to lifelong therapy in suitable cases [25].

### HMC testing strategy

**Fig. 1:**
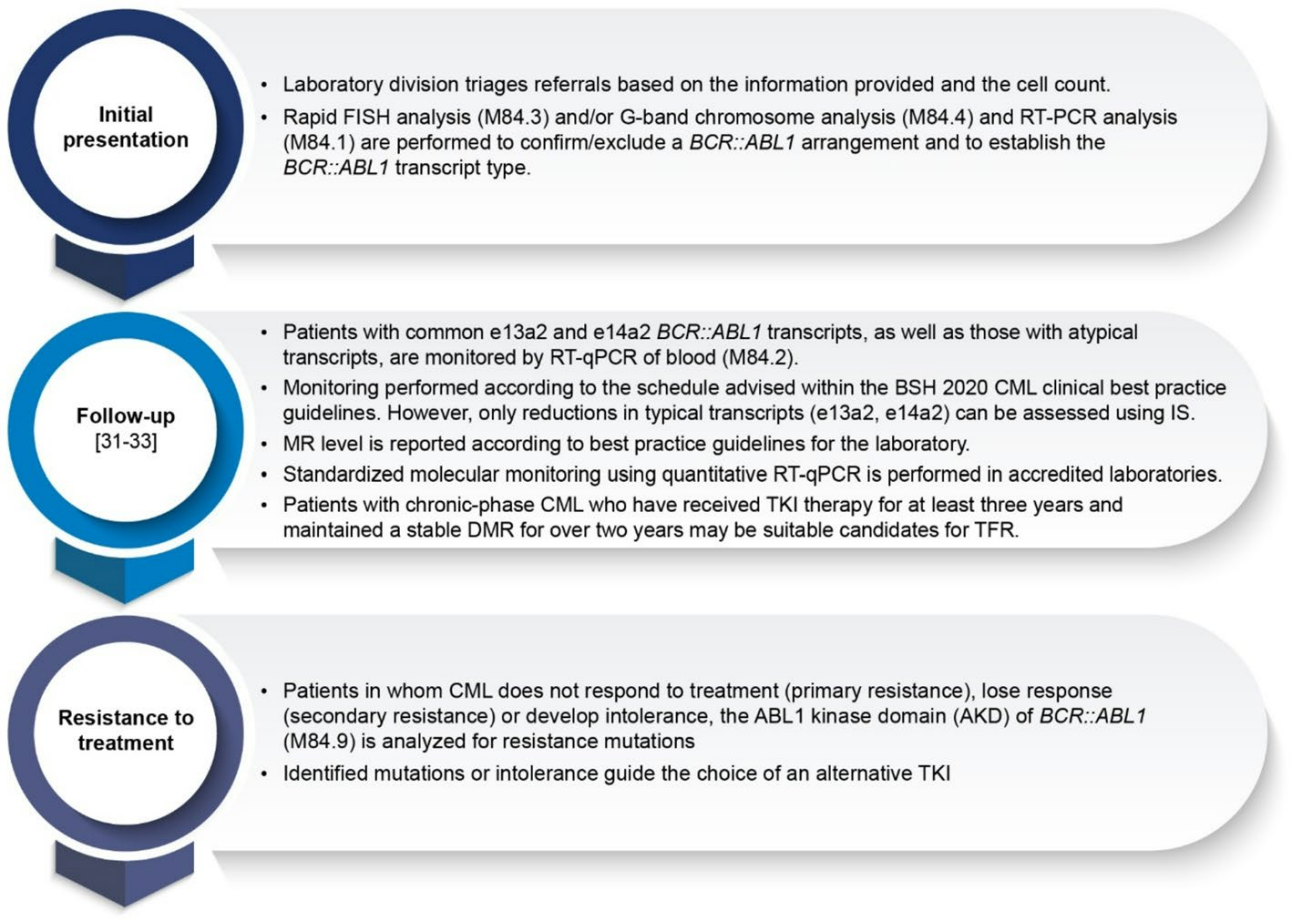
HMC testing strategy for CML: Diagnostic and monitoring workflow. *AKD* ABL 1 kinase domain, *BSH* British Society for Hematology, *CML* Chronic myeloid leukemia, *DMR* Deep molecular response, *FISH* Fluorescence in situ hybridization analysis, *IS* International scale, *MR* Molecular response, *RT-PCR* Reverse transcription polymerase chain reaction, *RT-qPCR* Reverse transcription quantitative polymerase chain reaction, *TFR* Treatment-free remission, *TKI* Tyrosine kinase inhibitor. M84.1, M84.2, M84.3, M84.4, and M84.9 codes refer to laboratory assay identifiers: M84.1—RT-PCR for *BCR::ABL1* transcript detection; M84.2—RT-qPCR for follow-up molecular monitoring; M84.3—rapid FISH analysis; M84.4—G-band chromosome analysis; M84.9—ABL1 kinase domain mutation analysis for TKI resistance

The HMC testing strategy ensures accurate diagnosis, standardized molecular monitoring, and timely detection of treatment resistance in CML patients. This approach aligns with international guidelines to optimize patient outcomes. The details of the HMC testing strategy are presented in Fig. 1 [31–33].

### Asciminib overview and pharmacology

Asciminib is indicated for the treatment of adult patients with newly diagnosed Ph-positive CML in CP (Ph + CP-CML), in patients previously treated for Ph + CP-CML, and in patients with Ph + CP-CML with T315I mutation [34]. It is a novel, first-in-class, Specifically Targeting the ABL Myristoyl Pocket (STAMP) kinase inhibitor. This unique mechanism of action differentiates it from other currently available TKIs, which target the adenosine triphosphate (ATP)-binding domain. Asciminib, the first approved allosteric inhibitor of *BCR::ABL1*, binds to a different site on the protein, potentially overcoming resistance to other TKIs, including that due to the T315I mutation [21]. The recommended dose for Ph + CP-CML is 80 mg once daily or 40 mg twice daily. The recommended dose for Ph + CP-CML with the T315I mutation is 200 mg twice daily.

Due to its distinct mechanism of action, mutations conferring resistance via the ATP binding site, including T315I, generally do not affect the efficacy of asciminib. However, mutations of the Src homology 1 (SH1) (outside the kinase domain) or Src homology 3 (SH3) domain could confer resistance to asciminib but not to ATP-competitive inhibitors [35, 36].

### Efficacy and safety of asciminib

The pivotal ASCEMBL phase III study (NCT03106779) compared asciminib (40 mg twice daily) with bosutinib (500 mg once daily) in 233 patients with CP-CML previously treated with two or more TKIs [37]. The study demonstrated that asciminib achieved a significantly higher MMR at 24 weeks (25.5%) than bosutinib (13.2%) (*p* = 0.029) [37]. By 96 weeks, the MMR rates increased to 37.6% with asciminib vs. 15.7% with bosutinib, suggesting a sustained long-term benefit [38]. Patients receiving asciminib also experienced a shorter median time to MMR (15.6 weeks) than with bosutinib (24 weeks), indicating a faster response to treatment [1]. Additionally, a higher complete cytogenetic response (CCyR) at 24 weeks was observed with asciminib (40.8%) than with bosutinib (24.2%), reinforcing its superior efficacy. Furthermore, 2-year progression-free survival (PFS) rates were 94.4% for asciminib and 91.1% for bosutinib, highlighting the durability of response with asciminib [37].

While efficacy endpoints are paramount, a thorough examination of asciminib’s safety profile is equally crucial, particularly considering the high comorbidity burden observed in the CML population [10]. The commonly reported adverse events (AEs) with asciminib included thrombocytopenia, neutropenia, diarrhea, nausea, rash, and vomiting. The incidence of grade ≥ 3 AEs was lower with asciminib (50.6%) than with bosutinib (60.5%). Additionally, treatment discontinuation due to AEs was significantly less frequent in the asciminib group (5.8%) than in the bosutinib group (21.1%), indicating better tolerability. Notable AEs associated with asciminib included myelosuppression, which was managed through dose adjustments or interruptions, and pancreatic toxicity, which primarily manifested as asymptomatic enzyme level elevations. Long-term follow-up data and results continue to emerge in patients with T315I mutations, further informing the clinical utility of asciminib in various CML patient populations [39].

Further, while TKIs have indeed improved the survival of CML patients, there are still issues associated with the impact of TKI treatment on QoL due to associated side effects [40]. In the phase 3 ASCEMBL trial, health-related quality of life was assessed using the EuroQol 5-Dimension 5-Level (EQ-5D-5 L) questionnaire, including the visual analogue scale (EQ-VAS), and remained stable, with a trend toward improvement in asciminib-treated patients. Using the Patient Global Impression of Change–CML, 47% of patients on asciminib reported improvement in CML symptoms vs. 20% on bosutinib, while symptom worsening was rare (< 4%). Additionally, patients on asciminib experienced greater reductions in activity impairment (6.5% vs. 1.0%), with similar trends in work productivity, highlighting a potential benefit of asciminib on daily functioning and overall QoL [41].

### Guideline recommendations

Given its Food and Drug Administration (FDA) and European approvals for adult patients with Ph + CP-CML previously treated with two or more TKIs, and for patients with T315I mutation, and now for first-line treatment, asciminib has been incorporated into international guidelines and treatment protocols for CML. Table 1 details various guidelines for the management of CP-CML [1, 20, 24, 32, 42–47].

**Table 1:** Guidelines on the management of CP-CML

Guidelines	Recommendations
NCCN Guidelines [24]	Asciminib is a preferred treatment option for any risk score in CML. For intermediate- or high-risk patients, second-generation TKIs and asciminib are preferred treatment options.
ELN [1, 20, 47]	The guideline recommends asciminib for newly diagnosed patients with CP-CML, for patients who have been previously treated with one TKI, and for patients who have been previously treated with two or more TKIs, with availability depending on local regulatory approvals.
ESMO Guidelines [42]	The guidelines recommend asciminib after two or more prior TKIs, noting its potential to overcome resistance mechanisms.
NICE, UK Guidelines [43]	The guidelines recommend asciminib as a cost-effective option after two or more TKIs.
BC Cancer Guidelines [44]	The guidelines include asciminib as a funded option for CML patients after failure of at least two TKIs.
EviQ (Australia) [45]	The guidelines recommend asciminib for adult patients with CP-CML previously treated with two or more TKIs.
French Recommendations [46]	Recent consensus recommends asciminib in therapy after two or more TKIs.
IQWIG, Germany [32]	Asciminib is recognized as an orphan drug, which highlights its significance in addressing unmet medical needs in CML.

*BC* British Columbia, *CML* Chronic myeloid leukemia, *CP-CML* Chronic phase-chronic myeloid leukemia, *ELN* European LeukemiaNet, *ESMO* European Society of Medical Oncology, *IQWIG* Institut für Qualität und Wirtschaftlichkeit im Gesundheitswesen, *NCCN* National Comprehensive Cancer Network, *NICE* National Institute for Health and Care Excellence, *TKI* Tyrosine kinase inhibitor

### Real-world evidence on asciminib

Several studies have provided real-world evidence for asciminib’s effectiveness, with high MMR (*BCR::ABL1* ≤ 0.1% IS) and molecular response grade 4 (MR4) (*BCR::ABL1* ≤ 0.01% IS) (Table 2) [31, 48–52]. While specific data for Qatar are limited, these studies provide valuable insights that may be applicable to the Qatari context [31, 48–52].

**Table 2:** Real-world evidence studies on asciminib

Country	MMR	MR4	Percentage discontinuation	Responses intolerant vs. resistance	Naïve vs. pre-treated	T315I: MMR rates
Spain [48]	26%–80%	15%–40%	28.5% (13.7 months)	58% vs. 28%	53% vs. 27%	NA
Russia [49]	29%–48%	17%–45%	17% (18 months)	75% vs. 23%	75% vs. 23%	*N* = 20; 48% vs. 29%
Italy [50]	44%	NA	NA	42% vs. 31%	46% vs. 23%	NA
UK [31]	53%	27%	39% (13 months)	75% vs. 29%	75% vs. 48%	*N* = 11; 63% vs. 43%
The Netherlands [51]	25%	12%	30% (8 months)	NA	25% vs. 10%	*N* = 12; 80% vs. 20%
Australia [52]	45%	NA	30% (14 months)	NA	NA	NA

*MMR* Major molecular response, *MR4* Molecular response grade 4, *NA* Not applicable

These real-world studies suggest that asciminib’s effectiveness and tolerability translate well to clinical practice, even in more diverse patient populations than those typically included in clinical trials.

### Clinical implications for Qatar based on guidelines and real-world evidence

Asciminib is approved in the European Union for treating adults with Ph + CP-CML previously treated with two or more TKIs. In 2024, the FDA approved asciminib for first-line treatment and beyond. It is also included in the ELN guidelines [1, 20]. The NCCN guidelines indicate asciminib as a treatment option for patients with CP-CML having the T315I mutation and/or previously treated CP-CML [24]. In Qatar, where patients often present at a young age, asciminib offers a favorable profile [6–8, 12]. It specifically targets the myristoyl pocket (STAMP) of the *BCR::ABL1* protein, which differentiates it from other ATP-binding TKIs. It also offers a better cardiovascular profile than ponatinib [53]. This makes asciminib a suitable option for patients with high cardiovascular risk, which is a common concern in Qatar [12, 15].

Further, the potential for achieving DMR with asciminib may contribute to increased opportunities for TFR attempts in eligible patients. This is particularly relevant for the younger CML population in Qatar, where successful TFR could significantly improve QoL and reduce the long-term economic burden of treatment [6–8]. However, it is important to note that data on TFR following asciminib treatment are still limited, and further research is needed to establish its role in TFR strategies.

### Cost-effectiveness considerations

**Fig. 2:**
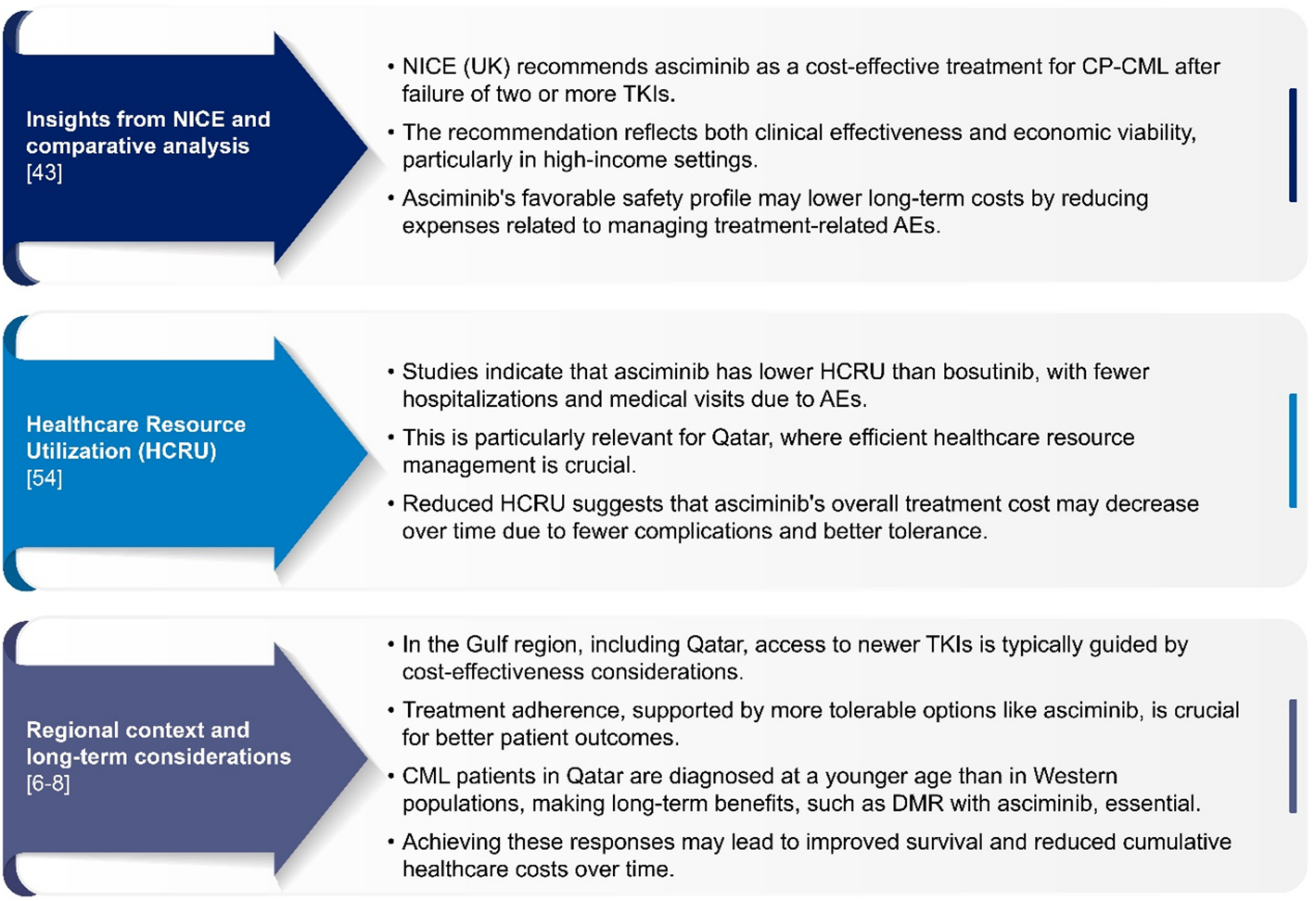
Cost-effectiveness and regional considerations for asciminib in CML treatment. *AE* Adverse event, *CML* Chronic myeloid leukemia, *CP-CML* Chronic phase-chronic myeloid leukemia, *DMR* Deep molecular response, *HCRU* Healthcare resource utilization, *NICE* National Institute for Health and Care Excellence, *TKI* Tyrosine kinase inhibitor, *UK* United Kingdom

The budget impact of CML treatments is an important factor in healthcare decision-making, especially in rapidly developing healthcare systems, such as Qatar’s. While direct cost-effectiveness data for asciminib specific to Qatar are not available, the annual direct treatment cost of asciminib is expected to be comparable to that of ponatinib. Insights into international assessments and regional considerations provide a relevant perspective for asciminib’s inclusion in the formulary of Qatar (Fig. 2) [6–8, 43, 54].

Beyond acquisition costs, asciminib offers potential financial advantages by reducing healthcare resource utilization and the economic burden associated with AEs [53, 54]. Ponatinib and bosutinib are associated with higher rates of cardiovascular, hepatic, and gastrointestinal toxicities, which often require intensive monitoring, additional outpatient visits, and, in severe cases, hospitalization [54, 55]. In contrast, asciminib has demonstrated a favorable safety profile, including lower rates of grade ≥ 3 cardiovascular events, leading to reduced monitoring requirements and lower toxicity management costs [48, 53, 54]. Given the high prevalence of cardiovascular comorbidities in the Qatari population [10, 11], which is also reflected in CML patients at baseline [12], a safer cardiovascular profile may lead to fewer AEs, ultimately translating into lower direct and indirect treatment-related costs.

Overall, the cost-effectiveness of asciminib, its reduced healthcare resource utilization (HCRU) compared with bosutinib’s HCRU, and its balance of efficacy and safety make it a promising option for patients with CP-CML in Qatar. These attributes align with the needs of the Qatari healthcare system, offering a potential for long-term cost savings and improved patient outcomes [8, 11, 12, 15, 53]. In addition, the drug acquisition cost is comparable between asciminib and ponatinib, the current third-line option used in Qatar, at the respective treatment doses [56], and its lower expected toxicity profile may provide a financial advantage over both ponatinib and other second-generation TKIs.

## Discussion

CML treatment has evolved significantly, yet for patients who are intolerant or resistant to currently available TKIs, the therapeutic needs remain unmet. While most CML patients respond well to first-line TKI imatinib, those requiring third-line therapy face a more complex and individualized treatment decision. In such settings, bosutinib is typically considered unless patients exhibit resistance or harbor the T315I mutation. Additionally, ponatinib or asciminib is also generally recommended in cases of resistance to prior TKIs, whereas another second-generation TKI can be considered in cases of intolerance. However, in Qatar, bosutinib is a non-formulary medication, leaving ponatinib as the usual option beyond second-line treatment. Its use, however, is limited by its cardiovascular toxicity, and current guidelines do not recommend it for patients with cardiovascular risk factors [9].

Initial studies have indicated that at the original 45 mg dose, ponatinib was associated with arterial occlusion and thrombosis in 20% of users [57]. However, the OPTIC trial demonstrated that a dose adjustment approach based on molecular response could help mitigate some of these risks. This approach, involving a starting dose of 45 mg and reducing it to 15 mg upon achieving ≤ 1% *BCR::ABL1* IS, was successful in meeting efficacy endpoints while improving the safety profile [58]. Despite these adjustments, cardiovascular risks remain a significant concern, particularly in Qatar, where cardiovascular comorbidities are highly prevalent [8, 12, 15]. This highlights the need for other treatment options with a more favorable cardiovascular safety profile for patients who have failed two or more lines of TKI therapy.

Emerging evidence supports the efficacy of asciminib in patients resistant or intolerant to multiple TKIs. The ASCEMBL study demonstrated that asciminib 40 mg twice daily achieved a greater MMR rate at 24 weeks than bosutinib 500 mg once daily (25.5% vs. 13.2%; *p* = 0.029) in 233 CML patients who had failed or were intolerant to at least two previous TKIs [37]. This efficacy was sustained at 96 weeks (37.6% vs. 15.8%) and was independent of the number of previous TKI lines (3rd, 4th, or 5th line) [38]. The secondary endpoint further supported these findings, showing a shorter median time to MMR, prolonged MMR duration, DMR (MR4 or better), and a higher rate of CCyR at week 24. The study is ongoing, and mature PFS and OS results are yet to be reported [37]. Asciminib demonstrated a more favorable safety profile than bosutinib, with lower incidence and severity of AEs, except for thrombocytopenia, and with less requirement for adjustments and treatment interruptions. The bosutinib dose used (500 mg), although authorized, may have presented tolerability issues, necessitating dose reductions [59, 60]. However, the size of the safety database was limited (*N* = 356), and longer-term safety data are not yet available. The primary endpoint (MMR at week 24) considers treatment failure due to lack of efficacy and discontinuation for any reason; therefore, the differences observed between asciminib and bosutinib could have been due to a higher proportion of discontinuations for reasons other than lack of efficacy in the bosutinib arm. However, the Committee for Medicinal Products for Human Use considers that the superiority of asciminib’s efficacy over bosutinib is supported by the key secondary endpoint (MMR at week 96) [1]. Asciminib’s efficacy, however, has not been directly compared with that of other third-line TKIs (nilotinib, dasatinib, or ponatinib). Notably, unlike bosutinib and ponatinib, asciminib has been studied in a randomized controlled clinical trial for third-line and subsequent-line treatment of CML.

Although theoretically, binding of asciminib to the myristoyl pocket of ABL could be effective against mutations in the ATP binding site, this has not been evaluated in the ASCEMBL trial. Patients with the T315I mutation who were treated with asciminib 200 mg BID were included in the phase I X2101 study; however, the European Medicines Agency did not consider these results in the evaluation of the efficacy of the drug [1]. Screening for mutations, such as the T315I mutation, is recommended in patients with a loss of response to treatment or at diagnosis for patients in the AP or BP [27, 28]. Mutational analysis enables optimization of individualized treatment in CML patients. Additional data on the efficacy of asciminib in patients with the T315I mutation will support the use of the drug in this set of patients.

Asciminib may offer better tolerability due to its lack of “off-target” kinase inhibition activity (such as proto-oncogene tyrosine-protein kinase, platelet-derived growth factor receptor, or KIT receptor tyrosine kinase) and could be useful following the occurrence of AEs caused by other TKIs (such as pleural effusion with dasatinib, diarrhea and liver toxicity with bosutinib, or cardiovascular events with nilotinib and ponatinib). Many of these complications can be avoided with appropriate patient selection and management at the lowest effective dose [61]. Asciminib’s safety profile is considered more favorable than ponatinib, despite a lack of direct comparison [1]. An indirect comparison highlighting the similar efficacy of asciminib and ponatinib, in the total cohort of patients and in the T315I mutation patient population, is shown in Table 3 [37–39, 58, 62]. However, as this comparison is not based on a head-to-head trial, differences in study design, patient populations, and other potential confounders should be considered when interpreting the findings. The treatment cost of both asciminib and ponatinib is comparable, with a more favorable safety profile for asciminib.

**Table 3:** Indirect comparison of asciminib and Ponatinib for CML patients and those harboring the T315I mutation

Parameters	Asciminib	Ponatinib
Efficacy		
Total population	ASCEMBL phase III	Phase II [58, 62]
MMR at 24 weeks (6 months) [37]	25.5% (for 40 mg BD dose) (ASCEMBL phase III)	54% (45 mg dose) (Phase II) [58, 62]
MMR at 96 weeks [38]	37.6% (for 40 mg BD dose) (ASCEMBL phase III)	Not reported
*BCR::ABL1* (IS) ≤ 1% at 36 months	Not reported	60%, 40%, and 40% (45 mg, 30 mg, and 15 mg cohorts, respectively) (OPTIC phase II) [58]
T315I mutation population	Phase I [39]	Phase II
MMR at 24 weeks (6 months)	57.9% (for 200 mg BD dose) (Phase I) [39]	70% (for 45 mg dose) (Phase II) [58, 62]
MMR at 96 weeks (22 months)	68.4% (for 200 mg BD dose) (Phase I) [39]	Not reported
*BCR::ABL1* ≤ 1% at 36 months	62.2% (Phase I) [39]	64%, 25%, and 16% (45 mg, 30 mg, and 15 mg cohorts, respectively) (OPTIC phase II) [58]
Safety		
Thrombocytopenia (Grade ≥ 3)	21.8% (ASCEMBL phase III) [37] 16.7% (Phase I) [39]	27% (OPTIC phase II) [58]
Neutropenia (Grade ≥ 3)	12.5% (Phase I) [39]	18% (OPTIC phase II) [58]
Hypertension (Grade ≥ 3)	8.4% (ASCEMBL phase III) [37]	9% (OPTIC phase II) [58]
Pancreatic toxicity (Grade ≥ 3)	1.1% (ASCEMBL phase III) [37]	Not reported
Anemia (Grade ≥ 3)	6.3% (Phase I) [39]	8% (OPTIC phase II) [58]
Arterial occlusion events	4.2% (Phase I) [39]	6% (45 mg), 6% (30 mg), and 4% (15 mg) (OPTIC phase II) [58]
Hypersensitivity (Grade ≥ 3)	0% (Phase I) [39]	Not reported
Myelosuppression (Grade ≥ 3)	20.8% (Phase I) [39]	Not reported

*BD* Twice daily, *MMR* Major molecular response, *OPTIC* Optimizing ponatinib treatment in CP-CML

While more data are needed on TFR outcomes, asciminib’s efficacy in achieving and maintaining MMR highlights its significant potential in patients with CML after two or more previous TKIs, including those with the T315I mutation, addressing an unmet therapeutic need beyond its authorized indication [37, 39].

### Position statement in Qatar

Given the epidemiology and comorbidity profile of the CML population, the use of asciminib as a third-line therapy addresses several unmet needs in CML management. The younger age at diagnosis [6–8], coupled with the high prevalence of CVDs and other metabolic disorders [12, 15], necessitates a treatment option that not only offers efficacy but also minimizes the risk of cardiovascular complications. Asciminib’s safety profile and efficacy data make it an important option for CML patients who have failed treatment or are intolerant to multiple TKIs, including those with the T315I mutation [37, 53]. The availability of asciminib in the national formulary will ensure that patients have access to the most up-to-date therapies in line with regional and international guidelines, including those from the Gulf Region Experts’ Survey, ELN, and NCCN [8, 20, 24].

In Qatar, asciminib should be considered for patients with CP-CML for whom two or more previous TKI treatments have failed and meet at least one of the following criteria: those harboring the T315I mutation (with preference over ponatinib in patients at cardiovascular risk), those with intolerance to previous TKIs, or those with resistance to prior treatment. Table 4 presents the suggested sequence of TKI treatment for CML patients in Qatar. Given its use, an evaluation of previous comorbidities, particularly a history of pancreatitis, is essential before initiating asciminib.

**Table 4:** Suggested sequence of TKI treatment in qatar’s patients for CP-CML

Line of therapy	TKI treatment	Considerations*
First line	First generation: imatinib OR Second generation: dasatinib or nilotinib	Selection should be based on goals of treatment and patient characteristics, including co-morbidities.
Second line	Second generation: dasatinib or nilotinib	If first-line treatment is with imatinib, selection should be based on comorbidities and mutation status; for patients with diabetes, dasatinib is preferred over nilotinib.
Third line	Third generation: asciminib or ponatinib	Selection should be based on comorbidities: for patients with cardiovascular risk, asciminib is preferred over ponatinib.

*Patient preference is always taken into consideration

*CP-CML* Chronic phase-chronic myeloid leukemia, *TKI* Tyrosine kinase inhibitor

## Conclusion

Asciminib is a preferred treatment option for CML in Qatar, with the ASCEMBL study demonstrating higher MMR rates than bosutinib at 24 and 96 weeks, along with shorter time to MMR, longer MMR duration, superior DMR (MR4 or better), and a higher CCyR rate at 24 weeks. It also has a more favorable safety profile, with fewer AEs and lower discontinuation rates. While long-term safety data, particularly on arterial occlusive events, are awaited, its inclusion in treatment guidelines expands options for CML patients with any risk scores. Future research should focus on direct comparisons with ponatinib, examining long-term outcomes, and utilizing real-world data to optimize personalized treatment strategies.

## Supplementary Information

Supplementary material 1.

## Data Availability

No datasets were generated or analysed during the current study.
