# Supplementary material for: Therapeutic positioning of asciminib in chronic myeloid leukemia patients previously treated with multiple tyrosine kinase inhibitors in Qatar
Source: Discov Oncol. 2025 Oct 23;16:1959. doi: 10.1007/s12672-025-03759-7 (PMC12550086; doi:10.1007/s12672-025-03759-7)
Supplement: Supplementary file 1 — Supplementary material 1. [file 12672_2025_3759_MOESM1_ESM.docx]

**Supplementary file**

**Journal Name:** Discover Oncology

**Therapeutic Positioning of Asciminib in Chronic Myeloid Leukemia Patients Previously Treated with Multiple Tyrosine Kinase Inhibitors in Qatar**

**Running Title**: Asciminib Positioning in CP-CML Treatment in Qatar

**Keywords**: Leukemia, myeloid, chronic phase; asciminib; tyrosine kinase inhibitors; cardiovascular diseases; safety; Middle East

Rola Ghasoub ^1^, Anas Hamad^1^, Susanna El Akiki^2^, Shehab Fareed^3^, Anil Ellahie^3^, Omar Ismael^3^, Mohamed A. Yassin^3^*

^1^Department of Pharmacy, National Center for Cancer Care and Research, Doha, Qatar

^2^Diagnostic Genomic Division, Hamad Medical Corporation, Doha, Qatar.

^3^Department of BMT & Hematology, NCCCR, Doha, Qatar

***Correspondence:**


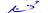


Mohamed A. Yassin,

Department of Hematology and BMT,
NCCCR, Doha, Qatar
E-mail: yassinmoha@gmail.com 
Phone: +974 - 55037393

**Table S1: Search strategy used for literature search**

Search limit: From 01 January 2010 to 31 December 2024

| **Search no.** | **Search string** |
| --- | --- |
| **PubMed** | |
| **#1** | "chronic myeloid leukemia"[All Fields] OR "leukemia, myelogenous, chronic, bcr abl positive"[MeSH Terms]  OR “CML”[All Fields] |
| **#2** | "asciminib"[All Fields] OR "STAMP inhibitor"[All Fields] |
| **#3** | "tyrosine kinase inhibitors"[All Fields] OR “TKI”[All Fields] OR "third-line therapy"[All Fields] OR “resistant CML”[All Fields] |
| **#4** | "cardiovascular risk"[All Fields] OR "comorbidities"[All Fields] |
| **#5** | "cost effectiveness"[All Fields] OR “healthcare resource utilization”[All Fields] |
| **#6** | "middle east"[MeSH Terms] |
| **#7** | #1 AND (#2 OR #3) AND #6 |
| **#8** | #1 AND (#2 OR #3) AND #4 AND #6 |
| **#9** | #1 AND (#2 OR #3) AND #5 AND #6 |
| **Embase** | |
| **#1** | 'chronic myeloid leukemia' OR 'leukemia, myelogenous, chronic, bcr abl positive'/exp OR CML |
| **#2** | asciminib OR 'STAMP inhibitor' |
| **#3** | 'tyrosine kinase inhibitors' OR TKI OR 'third-line therapy' OR 'resistant CML' |
| **#4** | 'cardiovascular risk' OR comorbidities |
| **#5** | 'cost effectiveness' OR 'healthcare resource utilization' |
| **#6** | 'middle east'/exp |
| **#7** | #1 AND (#2 OR #3) AND #6 |
| **#8** | #1 AND (#2 OR #3) AND #4 AND #6 |
| **#9** | #1 AND (#2 OR #3) AND #5 AND #6 |
| **Cochrane library** | |
| **#1** | "chronic myeloid leukemia" OR [mh "leukemia, myelogenous, chronic, bcr abl positive"] OR CML |
| **#2** | asciminib OR "STAMP inhibitor" |
| **#3** | "tyrosine kinase inhibitors" OR TKI OR "third-line therapy" OR "resistant CML" |
| **#4** | "cardiovascular risk" OR comorbidities |
| **#5** | "cost effectiveness" OR "healthcare resource utilization" |
| **#6** | [mh "middle east"] |
| **#7** | #1 AND (#2 OR #3) AND #6 |
| **#8** | #1 AND (#2 OR #3) AND #4 AND #6 |
| **#9** | #1 AND (#2 OR #3) AND #5 AND #6 |
